# Maternal Nutrition During Pregnancy and Fetal Outcome, Short- and Long-Term Health Effects: A Narrative Review

**DOI:** 10.3390/nu18091375

**Published:** 2026-04-27

**Authors:** Maria Elena Capra, Arianna Bellani, Martina Berzieri, Alessandra Fradusco, Susanna Esposito, Giacomo Biasucci

**Affiliations:** 1 Pediatrics and Neonatology Unit, Guglielmo da Saliceto Hospital, 29121 Piacenza, Italy; m.capra@ausl.pc.it (M.E.C.); g.biasucci@ausl.pc.it (G.B.); 2 Department of Medicine and Surgery, University of Parma, 43126 Parma, Italy; 3 Pediatric Clinic, University Hospital of Parma, 43126 Parma, Italy

**Keywords:** pregnancy, nutrition, offspring, non-communicable diseases

## Abstract

Over recent decades, a substantial body of research has expanded our understanding of how early-life conditions influence long-term health. These observations led to the formulation of the Barker Hypothesis, which postulates that adverse nutritional exposures during fetal life can induce persistent physiological and metabolic adaptations, thereby increasing susceptibility to chronic diseases later in life. This narrative review aims to provide a comprehensive overview of current recommendations for adequate maternal nutrition during pregnancy, with particular emphasis on key nutrients and specific dietary patterns. In addition, the effects of maternal diet on placental function and fetal growth are examined. A literature search was conducted in the following electronic databases: MEDLINE (via PubMed), Scopus, Web of Science, Embase, and the Cochrane Library. Manuscripts published between 2005 and 2025 were considered. The impact of prenatal nutritional exposures on immune development, neurodevelopment, metabolic regulation, and gut microbiota is also discussed, highlighting how these mechanisms may contribute to an increased long-term risk of non-communicable diseases, including obesity, metabolic syndrome, and neuropsychiatric disorders. Maternal nutrition during pregnancy plays a crucial role in shaping infants’ and children’s health, particularly regarding the development of non-communicable diseases. Therefore, ensuring adequate nutritional intake during this critical period—both quantitatively and qualitatively—is essential to optimize health outcomes for the newborn and to promote long-term well-being throughout childhood and beyond.

## 1. Introduction

Over recent decades, extensive research has expanded our understanding of how early-life conditions shape long-term health. The recognition that adult non-communicable diseases arise not only from genetic predisposition and postnatal lifestyle factors, but also from influences within the intrauterine environment, underpins the Developmental Origins of Health and Disease (DOHaD) paradigm [1,2]. Foundational insights into this concept were provided by the pioneering work of David J. P. Barker, who demonstrated strong associations between low birth weight and an increased risk of cardiovascular and metabolic diseases in adulthood [3,4,5,6,7]. These observations led to the formulation of the Barker Hypothesis, which proposes that adverse nutritional exposures during fetal life can induce persistent physiological and metabolic adaptations, thereby increasing susceptibility to chronic diseases later in life [2]. Maternal nutrition serves as a crucial component of the DOHaD framework, functioning as a key environmental factor that “programs” the long-term health outcomes of the fetus. According to this framework, nutrition prior to and during pregnancy shapes fetal growth patterns and metabolic environments, which in turn affects the likelihood of developing chronic non-communicable diseases (NCDs)—including obesity, type 2 diabetes, and cardiovascular disease—later in life.

**Figure 1:**
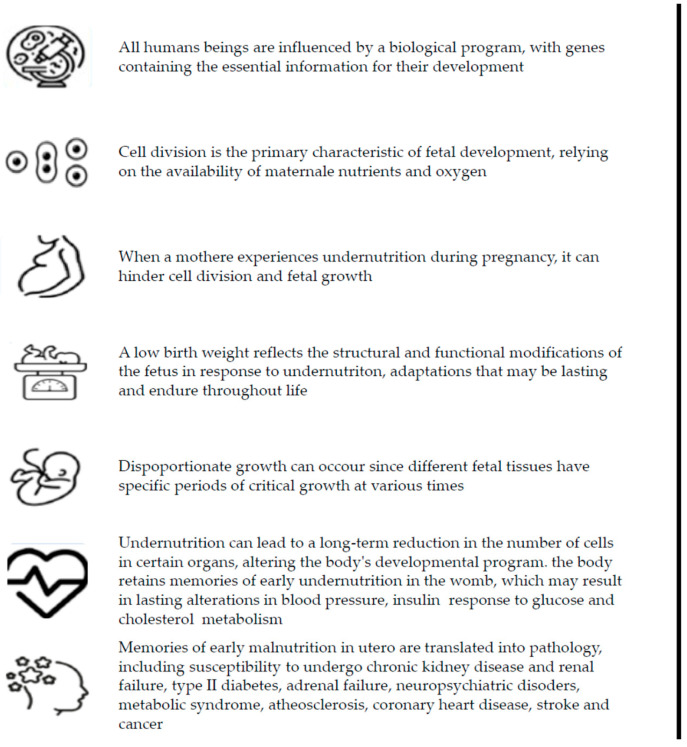
Main aspects of the Developmental Origins of Health and Disease (DOHaD) paradigm.

The main concepts underlying the DOHaD paradigm are summarized in Figure 1.

Further research has demonstrated that maternal nutrition plays a central role in shaping fetal growth, organ maturation, and long-term health outcomes. An adequate maternal diet—both in terms of quality and quantity—is essential to support normal fetal development and to reduce the risk of intrauterine growth restriction or excessive fetal growth [8]. Numerous studies have investigated the impact of specific nutrients and individual food items on fetal growth trajectories and birth outcomes [9,10,11,12]. However, over time, research has increasingly shifted from a reductionist focus on single nutrients toward the analysis of overall dietary patterns, which provide a more comprehensive assessment of maternal diet quality [13,14,15,16]. This transition reflects growing recognition that associations based on individual nutrients often show small effect sizes and may be confounded by complex biological interactions; in contrast, dietary pattern analyses more accurately capture real-world eating behaviors and their integrated physiological effects [17,18].

Importantly, maternal diet influences not only fetal growth but also developmental programming, partly through its effects on molecular mechanisms regulating gene expression [19]. Since the mid-2000s, accumulating evidence has shown that nutrition can act as a key epigenetic modulator. Nutritional exposures during critical windows of development can alter fundamental epigenetic processes, including DNA methylation, histone modifications, and non-coding RNA regulation [19,20,21,22]. These epigenetic modifications provide a mechanistic framework explaining how maternal dietary patterns—and the metabolic, hormonal, and inflammatory environments they generate—can exert long-lasting effects on organ development, metabolic homeostasis, and disease susceptibility. For instance, folate is involved in DNA methylation and cellular division and a deficiency of folate in mothers can result in genome-wide hypomethylation (reduced methylation), which is associated with neural tube defects and changes in metabolism in their children. N-3 Polyunsaturated Fatty Acids can influence the availability of methyl groups and are associated with improved DNA methylation patterns, especially in newborns of smoking mothers [23,24]. Nevertheless, despite significant advances in this field, many of the underlying biological pathways remain not fully understood.

A deeper understanding of how maternal nutrition, dietary patterns, and other modifiable factors influence fetal programming offers a valuable opportunity to develop preventive strategies to reduce the incidence and severity of non-communicable diseases from early life onward. Accordingly, this narrative review aims to provide a comprehensive overview of current recommendations for adequate maternal nutrition during pregnancy, with particular emphasis on key nutrients and specific dietary patterns. We aim to focus on the dyad mother–fetus and mother–child, instead of analyzing nutrition effects during pregnancy in two distinct ways. In addition, we summarize the effects of maternal nutrition on placental function and fetal growth. Finally, we discuss how prenatal nutritional exposures influence gut microbiota, immune system, neurodevelopment, and metabolic regulation, and how these mechanisms may increase the long-term risk of non-communicable diseases, including neuropsychiatric disorders, obesity, and metabolic syndrome.

## 2. Materials and Methods

A literature search was conducted in the following electronic databases: MEDLINE (via PubMed), Scopus, Web of Science, Embase, and the Cochrane Library. Publications published between 2005 and 2025 were considered. The search strategy included randomized controlled trials and systematic reviews. The following combinations of keywords were used: (“nutrition” AND “pregnancy”) OR (“nutrition” AND “fetal”) OR (“dietary patterns” AND “pregnancy”) AND (“newborn” OR “infant” OR “child”) AND (“non-communicable disease” OR “outcome index” OR “ birth weight” OR “weight excess” OR “failure to thrive” OR “ dyslipidemia” OR “neuropsychiatric disorders” OR “metabolic syndrome”).

No database-level filters were initially applied. The search was limited to full-text articles published in English. Eligible studies included reviews, meta-analysis, peer-reviewed, original research studies published in English that evaluated maternal or fetal outcomes in relation to nutritional status during any trimester of pregnancy. Exclusion criteria included manuscripts not available in English, studies published before 2005, and articles not belonging to the predefined study categories. This is a narrative review; therefore, no formal systematic selection or risk-of-bias assessment was performed.

To enhance comprehensiveness, the reference lists of selected articles were manually reviewed. Two independent reviewers screened titles and abstracts, followed by full-text assessment of potentially eligible studies. Discrepancies between reviewers were resolved through discussion and, when necessary, by consultation with a third reviewer.

As this is a selective narrative review, the final selection of studies focused on large, well-conducted randomized controlled trials, systematic reviews and meta-analysis, umbrella reviews and large, well-conducted narrative reviews addressing the role of maternal nutrition during pregnancy in shaping short- and long-term health outcomes in the offspring.

## 3. Recommendations for Adequate Maternal Nutrition During Pregnancy

Both National and International guidelines (elaborated by WHO, by the International Federation of Gynecologists and Obstetrics and by the Academy of Nutrition and Dietetics) [25,26,27,28,29,30] consistently emphasize that maintaining appropriate energy intake is essential to achieve the recommended gestational weight gain (GWG). They advocate for a nutrient-dense dietary pattern based on a wide variety of minimally processed foods, complemented—when necessary—by targeted micronutrient supplementation (such as folic acid, vitamin B12 or iron) tailored to specific population needs [25,26,27,28,29,30]. In addition, regular physical activity and healthy lifestyle behaviors, including abstinence from smoking and alcohol, are considered fundamental components of a healthy pregnancy [25,26,27,28,29,30,31,32].

Dietary counseling should be individualized according to pre-pregnancy body mass index (BMI), habitual dietary patterns, and socio-cultural context. Moreover, GWG and lifestyle factors should be regularly monitored throughout pregnancy to ensure optimal maternal and fetal outcomes [27,30,31]. Socioeconomic position (income, education, and occupation) and cultural elements (beliefs, customs, and family food priorities) have a significant influence on maternal diet as well. Reduced nutritional intake, increased use of processed foods, and poorer diets are frequently associated with lower income and education levels. Food choices are also greatly influenced by religious fasting and cultural customs. Income and purchasing power, education level, employment and role, and social support are examples of socioeconomic factors. Cultural beliefs and rituals, food distribution practices, food availability and advertising, and religious practices are examples of cultural and environmental factors [27,30,31].

### 3.1. Energy Requirements

Energy requirements increase modestly and progressively during pregnancy. According to the Italian LARN (Livelli di Assunzione di Riferimento per la popolazione Ita-liana, V revision), additional energy needs are approximately 70 kcal/day during the first trimester, 260 kcal/day during the second trimester, and 500 kcal/day during the third trimester [26]. A further increase of approximately 500 kcal/day is recommended during the first six months of breastfeeding. These values are consistent with other international guidelines [26,33]. Energy intake should be individualized based on pre-pregnancy BMI and physical activity level, to remain within recommended GWG ranges [25,27,34,35,36].

### 3.2. Macronutrients

Carbohydrates should provide 45–65% of total daily energy intake [26]. A minimum intake of 175 g/day is recommended to ensure adequate glucose supply for both maternal and fetal brain metabolism [25,26,29]. Whole grains should account for at least 50% of total carbohydrate intake, while simple sugars (mono- and disaccharides) should not exceed 10–15% of total energy intake and should be adjusted according to physical activity level. Simple sugars should be adjusted based on physical activity levels because they are rapidly digested, high-energy fuels that the body either uses immediately or stores as fat. Adjusting intake ensures that the body receives necessary energy during high-intensity training while avoiding the metabolic dysfunction, inflammation, and weight gain associated with excessive sugar intake during sedentary periods [25,27]. Consumption of at least five daily portions of fruits and vegetables is encouraged to ensure adequate fiber intake and improve postprandial glycemic control [26,37].

Protein plays a critical role during pregnancy, contributing to the development of structural tissues and the synthesis of enzymes, transport proteins, and hormones [38]. Protein sources include plant-based foods (legumes, grains, nuts), animal-derived products (meat, dairy), and alternative sources such as algae and fungal-derived mycoproteins [39]. Protein quality is evaluated using the Protein Digestibility-Corrected Amino Acid Score (PDCAAS). Animal-derived proteins typically achieve scores close to 1 due to their complete essential amino acid profile, whereas most plant-based proteins score below 0.7 [40]. However, combining complementary plant proteins can improve overall protein quality [41]. Protein intake during pregnancy should remain within 25% of total energy intake [37]. Balanced energy–protein supplementation has been associated with reduced risk of stillbirth, low birth weight (LBW), and small for gestational age (SGA) infants across different income settings [8]. After the first trimester, protein requirements increase to approximately 1.1 g/kg/day to support the growth of maternal and fetal tissues [38]. As for practical recommendations, daily intake should increase by approximately 1 g/day in the first trimester, 8 g/day in the second trimester, and 26 g/day in the third trimester compared with baseline adult requirements [26,42].

Total fat intake should account for 20–35% of total daily energy, as for non-pregnant women [28,29,33]. However, fat quality is of primary importance. Pregnant women should prioritize unsaturated fats (found in fish, plant oils, nuts, and seeds) and limit saturated fats and trans fats [25]. Essential fatty acids, including linoleic acid, alpha-linolenic acid, and long-chain polyunsaturated fatty acids (PUFAs) such as arachidonic acid (AA), eicosapentaenoic acid (EPA), and docosahexaenoic acid (DHA), are fundamental structural components of cellular membranes and are crucial for fetal tissue development [43]. Maternal levels of these fatty acids decline during pregnancy; therefore, adequate intake is necessary to meet both maternal and fetal needs [37,44]. DHA requirements increase by approximately 100–200 mg/day during pregnancy [25]. To balance nutritional benefits with safety, the Environmental Protection Agency and the Food and Drug Administration (FDA) recommend choosing low-mercury fish (e.g., salmon, shrimp, cod) while avoiding species with high mercury content (e.g., shark, swordfish, king mackerel) [45]. Supplementation with n-3 long-chain PUFAs has been associated with reduced risk of preterm birth, early preterm birth, and low birth weight, as highlighted in a 2018 Cochrane review [46]. The observed rise in the incidence of childhood atopic disease may be explained by the shift in the Western diet from a relative balance between pro-inflammatory omega-6 fatty acids and anti-inflammatory omega-3 fatty acids to a diet where omega-6 fatty acids are overwhelmingly predominant [47,48,49]. Studies on humans and animals have demonstrated that consumption of omega-3 fatty acids inhibits cell-mediated immune responses [47,48,49].

In particular, we have previously demonstrated that supplementing with fish oil high in docosahexaenoic acid (DHA) and eicosapentaenoic acid (EPA) during pregnancy changed Th2/Th1 chemokine ratios at birth, decreasing the mostly Th2 response that has been linked to allergy illness. However, evidence regarding long-term child developmental outcomes remains inconclusive. While universal supplementation may be reasonable, a more targeted strategy may be preferable as further evidence emerges.

### 3.3. Micronutrients

Although a balanced diet can meet most micronutrient requirements, several nutrients have increased needs during pregnancy and are prioritized in international guidelines, including folate, iron, iodine, calcium, vitamin D, vitamin A, and selected trace elements [25,27,29].

Folate is essential for DNA and RNA synthesis, homocysteine methylation, and amino acid metabolism [50,51,52]. Requirements increase during the periconceptional period due to rapid cellular proliferation and neural tube development [48,49,50]. Supplementation reduces the risk of neural tube defects, maternal anemia, and low birth weight [52,53], and may also lower the risk of congenital heart defects [9,54]. Guidelines recommend 600 mcg of synthetic folic acid daily during pregnancy (400 mcg is recommended daily for women of child-bearing age before conception) [25,26,27,29,32]. Women at high risk of neural tube defects are advised to take 4–5 mg/day beginning preconceptionally [32,55].

Iron is essential for hemoglobin synthesis and oxidative metabolism [56]. Requirements increase during pregnancy to support expanded maternal and fetal red cell mass. Iron deficiency has been associated with impaired fetal growth, LBW, and preterm birth [56], and possibly long-term cardiometabolic effects [11]. Routine supplementation is debated. NICE (National Institute for Health and Care Excellence) and SINU (Società Italiana di Nutrizione Umana) do not recommend universal supplementation [55,56], whereas WHO (World Health Organization) and FIGO (Federazione Italiana Ginecologi e Ostetrici) advise 30–60 mg/day of elemental iron [27,57]. Iron-deficiency anemia (Hb < 11 g/dL in the first trimester; <10.5 g/dL after 28 weeks) should be treated with 60–120 mg/day of oral iron [25,58,59,60]. Dietary counseling should emphasize heme iron sources and strategies to enhance non-heme iron absorption (e.g., vitamin C intake) while limiting inhibitors such as tea, coffee, and calcium-rich foods [25,27,61].

Calcium is essential for fetal skeletal development. The recommended intake during pregnancy is 1.1 g/day [26]. WHO recommends daily calcium supplementation of 1.5–2.0 g for pregnant women with low dietary calcium intake to prevent pre-eclampsia, particularly starting from 20 weeks gestation. Supplements are generally safe but may cause constipation, while high-calcium diets (dairy, leafy greens) are preferred to ensure adequate intake for both mother and fetus. Adequate calcium intake during pregnancy is essential for fetal bone development and maternal health, with a recommended daily intake of 1000–1300 mg/day for adults and pregnant teens [25,27,29,30].

Vitamin D status depends largely on endogenous synthesis influenced by sun exposure and individual factors [62,63,64,65,66,67]. Deficiency has been associated with preterm birth, SGA, gestational diabetes, and miscarriage [68]. Recommendations vary: FIGO and NICE suggest 400 IU/day for all pregnant women [29,60], WHO recommends 200 IU/day in suspected deficiency [69], while some Italian societies recommend selective supplementation [25].

Vitamin A supplementation is recommended only in populations with documented deficiency or in HIV-positive women, as routine supplementation is not advised [37,70,71,72].

Vitamin B12 is essential for one-carbon metabolism and neurodevelopment [73,74,75]. Although deficiency is associated with adverse outcomes [76,77,78,79], routine supplementation is not recommended unless deficiency is present or risk factors exist (e.g., vegetarian or vegan diets) [25,61].

Iodine is critical for thyroid hormone production and fetal brain development [80]. EFSA (European Food Safety Authority) and LARN recommend 200 µg/day [26,81], while WHO suggests a total intake of approximately 250 µg/day in pregnancy [82,83]. Supplementation (150–250 µg/day) is recommended in areas of mild-to-moderate deficiency, ideally beginning preconceptionally [84,85].

A balanced diet generally provides sufficient zinc, magnesium, selenium, copper, and vitamins B6, C, and E; routine supplementation is not recommended [25,86].

### 3.4. Alcohol and Caffeine

Alcohol should be avoided during pregnancy due to its association with fetal alcohol spectrum disorders (FASD), miscarriage, preterm birth, and SGA infants [25,37].

Although evidence regarding caffeine remains inconclusive, intake above 200 mg/day has been associated with increased risk of pregnancy loss and low birth weight. Caffeine is found in tea, energy drinks, chocolate, and soda. Therefore, limiting caffeine consumption to ≤200 mg/day is generally recommended [25,27].

Pregnancy-related over-supplementation, which is frequently brought on by taking too many vitamins or in excess, can have serious negative effects on both the mother and the fetus, including birth abnormalities, metabolic problems, and neurodevelopmental disorders. High levels of iron, folic acid, and vitamin A are linked to the most dangerous dangers. Birth abnormalities, especially cleft lip, palate, and other deformities, can result from an overabundance of vitamin A. Overconsumption of folic acid has been linked to increased body fat, possible neurodevelopmental problems, and impaired cognitive development in kids. It can also raise the risk of gestational diabetes. Complications including gestational diabetes and preterm birth might result from high iron dosages [87].

Excessive macronutrient intake can be dangerous as well. For example, maternal overnutrition (excess calories, high sugar/fat diet) can induce fetal hyperinsulinemia and adiposity. In situations of undernutrition, the placenta may limit the transport of nutrients, either through a reduction in size or changes in the expression of transporters, while the fetal endocrine systems, such as the hypothalamic centers for appetite, modify themselves to deal with the lack of resources. In cases of overnutrition, like maternal obesity or gestational diabetes mellitus, elevated levels of glucose and insulin in the fetus lead to an increase in fat accumulation during pregnancy. Both of these conditions result in epigenetic modifications, such as DNA methylation and histone alterations, that permanently affect gene expression in the developing fetus [88].

The main dietary recommendations during pregnancy are summarized in Table 1.

## 4. Dietary Patterns in Pregnancy

Traditional dietary models, particularly the Mediterranean diet, are widely recommended during pregnancy, due to their well-documented benefits for both maternal and offspring health. However, growing interest in alternative dietary approaches, including vegetarian, vegan, ketogenic, and other restrictive patterns, requires careful evaluation of their nutritional adequacy and safety during pregnancy [89].

### 4.1. Mediterranean Diet

The Mediterranean diet (MedDiet) is characterized by high daily intake of fruits, vegetables, legumes, whole grains, nuts, and olive oil; moderate consumption of dairy products, fish, and poultry; and limited intake of red and processed meats, refined sugars, and highly processed foods [90].

Extensive evidence supports adherence to the MedDiet during pregnancy, as it generally meets gestational nutritional requirements and has been associated with a reduced risk of gestational complications [91]. Moreover, adherence to a Mediterranean dietary pattern has been linked to lower rates of low birth weight and intrauterine growth restriction, as well as favorable gestational age at delivery [92,93]. Higher MedDiet adherence scores have also been associated with reduced odds of atopy and lower incidence of bronchiolitis in offspring [94,95]. Moreover, adherence to a Mediterranean dietary pattern has been linked to improved verbal intelligence and executive function [95]. Overall, MedDiet represents a balanced and evidence-based dietary model for pregnancy.

### 4.2. Western-Style Diet

The Western dietary pattern is typically characterized by high consumption of red and processed meats, refined grains, sugar-sweetened beverages, salty snacks, high-fat dairy products, and ultra-processed foods, alongside low intake of fruits and vegetables [95,96].

This dietary pattern is generally considered nutritionally suboptimal during pregnancy, as it may fail to meet increased micronutrient requirements essential for normal fetal development [97]. Western-style diets have been associated with a higher risk of SGA infants, lower birth weight, and increased incidence of preterm birth [97,98,99]. Additionally, associations with offspring forearm fractures have been reported [100], as well as higher rates of allergic diseases and asthma, potentially related to reduced intake of n-3 polyunsaturated fatty acids [101]. These findings suggest that a Western dietary pattern may adversely affect both short- and long-term offspring health.

### 4.3. Plant-Based Diets

Plant-based diets (PBDs) encompass a spectrum of eating patterns characterized by reduced or complete exclusion of animal-derived foods, with vegan diets representing the most restrictive form [102]. These dietary patterns are increasingly adopted for ethical, environmental, and health-related reasons, including during critical developmental periods [103].

While well-planned PBDs may confer metabolic and cardiovascular benefits, their safety during pregnancy remains debated. Evidence suggests that, although potentially beneficial, PBDs may pose risks related to fetal growth and birth outcomes, though findings are inconsistent [102,104,105,106,107]. According to the Academy of Nutrition and Dietetics, appropriately planned and supplemented vegetarian and vegan diets can be considered safe throughout the life cycle, including pregnancy [108].

However, careful monitoring is essential. PBDs have been associated with increased risk of deficiencies in iron, vitamin B12, vitamin D, calcium, iodine, and potentially protein and omega-3 fatty acids [105,109]. To mitigate these risks, tailored dietary counseling and appropriate supplementation are crucial. Emphasis should be placed on fortified foods and plant-based sources of key nutrients, such as legumes, nuts, seeds, and algae-derived omega-3 fatty acids, to support optimal maternal and neonatal outcomes [104].

Pros and cons of vegan dietary pattern during pregnancy are summarized in Table 2.

### 4.4. Ketogenic Diet

The ketogenic diet (KD) is characterized by high fat, moderate protein, and very low carbohydrate intake, inducing a state of nutritional ketosis [110]. Its safety during pregnancy remains largely uncertain.

In specific metabolic conditions, such as Pyruvate Dehydrogenase Complex deficiency or Glut1 deficiency syndrome, maternal ketogenic therapy has been associated with positive fetal outcomes [111]. However, animal studies suggest that maternal KD may alter fetal growth trajectories, impair brain development, and induce structural and behavioral changes potentially linked to anxiety and depressive phenotypes later in life [111,112,113]. Furthermore, excessive fetal exposure to lipids may promote overgrowth and increased adiposity, which are predictors of childhood obesity and metabolic disorders [114,115].

Given the limited human data and potential risks, most reviews recommend avoiding carbohydrate-restrictive or ketosis-inducing diets during pregnancy unless medically indicated and carefully supervised [41,116,117].

### 4.5. Gluten-Free Diet

A gluten-free diet (GFD) excludes wheat, barley, and rye and is the cornerstone of treatment for celiac disease [118]. It may also be prescribed for other gluten-related conditions. Permitted foods include meat, dairy, fruits, vegetables, and naturally gluten-free grains such as rice, corn, quinoa, and amaranth [89].

Because many gluten-containing foods are important sources of essential nutrients, individuals following a GFD may be at risk of deficiencies in iron, folic acid, calcium, magnesium, vitamin D, zinc, and fiber. Adequate intake of alternative gluten-free sources—including leafy greens, legumes, nuts, dairy products, fortified foods, and naturally gluten-free whole grains—is therefore essential [89].

For pregnant women with celiac disease, strict adherence to a GFD significantly reduces the risk of miscarriage, preterm birth, low birth weight, intrauterine growth restriction, and certain congenital anomalies [119,120,121]. However, in women without celiac disease, gluten avoidance offers no proven benefit and may unnecessarily increase the risk of nutritional deficiencies [122]. Nutritional monitoring is therefore recommended when a GFD is followed during pregnancy.

### 4.6. Intermittent Fasting

Intermittent fasting (IF) focuses on meal timing rather than food composition. The most common approach involves restricting food intake to an 8 h window and fasting for the remaining 16 h, during which only water or unsweetened beverages are consumed [123].

While IF may improve insulin sensitivity, glucose metabolism, and inflammatory markers—particularly in women with polycystic ovary syndrome (PCOS) [89]—its safety during pregnancy is uncertain. Evidence suggests potential risks, including lower birth weight, reduced amniotic fluid volume, and increased risk of preterm birth. Therefore, IF should be approached with caution and only under close medical supervision during pregnancy [89,124].

## 5. Ultra-Processed Food Consumption During Pregnancy

Ultra-processed foods (UPFs) are industrial formulations composed of refined ingredients, including sugars, fats, oils, and salt, combined with additives designed to enhance palatability, shelf-life, and sensory characteristics [125,126]. The increasing consumption of UPFs among women of reproductive age has prompted growing concern regarding their impact on pregnancy and offspring health [127,128].

Maternal consumption of UPFs during pregnancy may influence fetal growth and development. Unhealthy dietary patterns have been associated with lower birth weight and a trend toward increased preterm birth [15]. Higher maternal UPFs intake has also been linked to increased neonatal adiposity [129,130]. However, a recent meta-analysis reported no significant association between unhealthy dietary patterns and birth weight, highlighting inconsistencies in the literature [17].

Emerging evidence suggests that high maternal UPFs consumption may adversely affect fetal brain development and later cognitive function [131]. The third trimester appears particularly vulnerable due to rapid central nervous system maturation [127,132]. Diets high in saturated fats may promote oxidative stress and neuroinflammation [133]; certain food additives, including bisphenols and nanoparticles, may cross the placental and blood–brain barriers, potentially interfering with neurodevelopment and increasing risk of behavioral disorders [131,134,135].

Associations between maternal UPFs intake and reduced verbal functioning in early childhood have been reported [128]. Similarly, diets rich in processed foods and added sugars have been negatively associated with cognitive development [136]. Trans fatty acids may further impair neuronal communication and memory processes [131,135].

Beyond neurodevelopment, prenatal exposure to UPFs may influence immune-related outcomes. Healthier maternal dietary patterns have been associated with reduced risk of infant atopic dermatitis [137]; higher UPFs intake has been linked to increased risk of atopic dermatitis in infancy [138] and food allergies [139]. However, findings are not entirely consistent, as some studies suggest potential protective associations between certain Western dietary patterns and wheeze [140].

Overall, although evidence is accumulating, further high-quality prospective studies to clarify the impact of maternal UPFs consumption on short- and long-term offspring health are needed.

## 6. Maternal Diet and Fetal Growth

### 6.1. Consumption of Highly Processed Food by the Mother—Impact on Fetal Health

Fetal growth—often assessed through deviations in birth weight such as small for gestational age (SGA) and large for gestational age (LGA)—is a key indicator of maternal–fetal health. Infants born SGA (birth weight <10th percentile for gestational age) or LGA (>90th percentile) face increased perinatal complications and long-term risks, including cardiometabolic morbidity and altered metabolic programming [141].

Maternal nutritional status profoundly influences placental development and function, including the expression of nutrient transporters, thereby shaping fetal growth trajectories. These data have been proved in animal studies and confirmed in human. Experimental evidence indicates that moderate maternal caloric restriction can induce uteroplacental insufficiency and alter placental transporter profiles; in murine models, reduced uterine blood flow was accompanied by differential regulation of the glucose transporter GLUT3 and the amino acid transporter LAT2 [142]. Consistently, models of maternal undernutrition and overnutrition demonstrate placental structural and vascular remodeling. In a sheep model of restricted or excessive feeding, angiogenic factors, chemokines, and signaling molecules showed marked changes at both gene and protein levels, suggesting that an adverse maternal diet can disrupt placental growth and nutrient-delivery architecture [143].

Maternal diet also modulates placental nutrient-sensing and endocrine signaling pathways, including glucose (GLUT1/GLUT3) and amino acid transport systems (SNATs, LATs), metabolic sensors such as mTOR, and insulin/IGF-1-related axes that coordinate maternal nutrient availability with fetal demand. In human placental tissue, maternal hyperglycemia and diabetes have been associated with increased phosphorylation of placental insulin-like growth factor-1 receptor (IGF-1R), supporting a link between altered maternal metabolism, enhanced placental nutrient signaling, and potential fetal overgrowth [144]. Micronutrient deficiencies, including vitamin D, folate, vitamin B12, and iron, have likewise been associated with impaired placental function in clinical and experimental studies, through mechanisms such as altered trophoblast proliferation, increased oxidative and inflammatory stress, and reduced nutrient-transporter expression, which may contribute to fetal growth restriction [145].

Beyond overall dietary patterns, specific maternal metabolic markers may influence the risk of fetal growth extremes. In a Japanese cohort, both low and high maternal total cholesterol in mid-pregnancy were associated with increased risks of SGA and LGA, respectively, suggesting that maternal lipid metabolism—and related factors such as diet and adiposity—may contribute to deviations in fetal growth [146]. Similarly, higher dietary fiber intake, lower glycemic load, and adequate essential fatty acids (e.g., DHA and EPA) have been associated with favorable pregnancy outcomes. However, one observational study in healthy women reported an approximately twofold higher risk of SGA among those following a low-glycemic index diet, raising the possibility that excessive restriction of glycemic load may reduce fetal nutrient availability in some contexts. This highlights the importance of individualized dietary recommendations: a low-GI approach may be beneficial in women with gestational diabetes or higher BMI but may be less appropriate in lean women at risk of SGA [37].

In this framework, maternal nutrition before and during pregnancy plays a crucial role in determining fetal growth and birth weight. Imbalanced dietary patterns, inadequate intake of essential nutrients, or excessive consumption of added sugars can negatively affect the intrauterine environment and limit nutrient delivery to the fetus, increasing the likelihood of adverse neonatal outcomes [147]. In the Brazilian ProcriAr cohort, Teixeira et al. showed that pre-pregnancy dietary patterns were associated with neonatal birth size, supporting the concept that maternal nutrition even before conception may influence fetal growth and early-life outcomes, and suggesting a potential protective role of diets centered on traditional and minimally processed foods [147]. In line with these findings, Abdollahi et al. reported in a systematic review and meta-analysis that healthy dietary patterns—characterized by fruits, vegetables, whole grains, legumes, and fish—were associated with reduced risks of SGA and preterm birth, whereas Western-style patterns were linked to increased risk of SGA and adverse outcomes including preeclampsia and macrosomia [17].

Conversely, Western-style diets rich in refined carbohydrates, added sugars, saturated fats, and ultra-processed foods have been associated with excessive fetal growth and higher rates of LGA infants. These patterns may promote maternal hyperglycemia, insulin resistance, and dyslipidemia, which can increase fetal nutrient exposure, adiposity, and macrosomia [148]. High intake of simple sugars—particularly from sugar-sweetened beverages and ultra-processed foods—has emerged as a key contributor to excessive gestational weight gain and metabolic complications. Several studies have linked frequent consumption of sugary foods and beverages to increased risk of gestational diabetes mellitus, hypertensive disorders, and fetal overgrowth leading to macrosomia [149,150]. Proposed mechanisms include systemic inflammation, oxidative stress, and placental dysfunction affecting nutrient transport and growth regulation; moreover, both glucose and fructose can cross the placenta and alter placental metabolism, potentially influencing fetal adiposity and development [149]. Beyond immediate birth outcomes, excessive maternal sugar intake has been associated with long-term offspring risks, including childhood obesity, metabolic dysregulation, and neurodevelopmental alterations, consistent with intrauterine programming mechanisms [150]. Collectively, these data support limiting added sugars during pregnancy and promoting balanced dietary patterns to optimize maternal metabolic status and fetal growth [150].

In addition to dietary patterns, adequacy of specific nutrients remains important. Sufficient intake of essential fatty acids (particularly DHA and EPA) supports neurodevelopment and influences fetal growth, whereas inadequate intake of protein and micronutrients such as iron, zinc, and folate is associated with fetal growth restriction. In contrast, excess intake of added sugars and ultra-processed foods may dysregulate maternal metabolism and increase the likelihood of both SGA and LGA outcomes depending on the maternal metabolic context [17].

Maternal nutrition may also affect the timing of delivery. Both undernutrition and overnutrition have been associated with altered gestational length, potentially increasing risks of preterm or post-term birth. Observational studies suggest that inadequate energy or micronutrient intake—particularly protein, iron, and vitamin D—may impair placental function, disrupt endocrine signaling (including progesterone and placental corticotropin-releasing hormone), and increase systemic inflammation, thereby activating pathways that promote earlier parturition [3,151]. Conversely, excessive energy intake or high-glycemic diets have been associated with prolonged gestation and increased risk of post-term birth, potentially mediated by altered fetal growth trajectories, placental nutrient sensing, and insulin/IGF signaling [152]. Animal studies further indicate that maternal protein-energy restriction and high-fat feeding can modify parturition timing through changes in placental CRH expression and prostaglandin synthesis, underscoring the placenta as a central mediator linking maternal nutritional status to gestational duration [153].

### 6.2. Maternal Plant-Based Diet and Fetal Growth and Timing of Delivery

Evidence suggests that predominantly plant-based dietary patterns during pregnancy may influence birth weight, although effects vary by population, dietary composition, and baseline nutritional status. In a Canadian multiethnic cohort, adherence to a plant-based dietary pattern was associated with lower birth weight and increased odds of SGA among white European mothers, alongside a reduced risk of LGA; in contrast, among South Asian mothers, the same pattern was associated with slightly higher birth weight, highlighting potential ethnic and contextual modifiers [154]. Similarly, a prospective study in China reported that a carbohydrate-rich plant-based diet—characterized by higher intake of foods such as potatoes—was associated with an increased risk of macrosomia [155].

Evidence linking plant-based diets to gestational length remains limited. In pregnant women with chronic kidney disease, adherence to a moderately protein-restricted plant-based diet was associated with a lower combined risk of preterm delivery (<37 weeks) or SGA infants. Although direct causal inference is difficult, these findings suggest that diet-related changes in maternal metabolic milieu may influence both fetal growth and parturition pathways [156]. Mechanistically, plant-based dietary patterns may affect placental nutrient transport, maternal glucose and lipid metabolism, and endocrine signaling pathways such as IGF-1 and leptin, thereby modulating fetal growth and possibly the timing of delivery [16]. Overall, outcomes appear to depend on dietary quality, adequacy of key nutrients, maternal BMI, and population-specific factors, underscoring the need for well-designed prospective studies [154,155,156].

### 6.3. Maternal Smoking During Pregnancy and Neonatal Outcomes

Maternal smoking is a major preventable risk factor for adverse perinatal outcomes, with consistent evidence linking tobacco exposure to fetal growth restriction and preterm birth. In a Swiss cohort, maternal smoking was associated with an adjusted odds ratio of approximately 2.1 for SGA and about 1.4 for preterm birth (<37 weeks) compared with non-smokers [157]. A U.S. study reported that even non-daily or low-intensity daily smoking during the third trimester increased the risk of SGA by approximately 1.4-fold, while the association with preterm birth appeared stronger among daily smokers [158].

Mechanistically, nicotine and carbon monoxide reduce placental perfusion and oxygen delivery (through increased carboxyhemoglobin), induce fetal hypoxia, and promote placental vascular remodeling consistent with uteroplacental insufficiency [157,159]. These pathways contribute to growth restriction and may activate inflammatory or stress-related signaling that promotes earlier parturition. Conversely, maternal overweight may increase LGA risk through increased nutrient availability, insulin resistance, and altered placental nutrient transport. When multiple exposures coexist (e.g., smoking and overweight), they may generate competing biological signals, emphasizing that simple birth-weight thresholds may not fully capture the complexity of fetal growth regulation and perinatal risk [160].

## 7. Maternal Nutrition During Pregnancy and Long-Term Effects on the Offspring

Maternal dietary habits before and during pregnancy influence not only intrauterine fetal growth but also long-term health outcomes in the offspring [8]. This concept is central to the developmental origins of health and disease paradigm: optimizing maternal nutrition and correcting macro- and micronutrient deficiencies may reduce the future risk of chronic non-communicable diseases (NCDs) by modulating mechanisms of fetal programming that shape health trajectories across the lifespan [161].

### 7.1. Effects of Maternal Nutrition on Neurodevelopment

Maternal nutrition during pregnancy plays a pivotal role in fetal brain development through multiple biological pathways [162]. Overall diet quality and adequate macronutrient intake provide essential substrates for neuronal proliferation, differentiation, and synaptogenesis, while micronutrients regulate critical processes such as DNA synthesis, methylation, myelination, and neurotransmitter production [163].

The growing adoption of PBDs warrants particular attention. While well-planned PBDs may reduce the risk of gestational complications such as preeclampsia and gestational diabetes, poorly balanced regimens may result in deficiencies of nutrients essential for neurodevelopment, including omega-3 fatty acids, iron, zinc, iodine, and vitamin B12 [164].

Vitamin B12 is fundamental for one-carbon metabolism and DNA methylation, processes crucial for neuronal maturation. Deficiency has been associated with impaired neurocognitive development, potentially mediated by DNA hypomethylation and altered gene expression [76,165]. A recent Cochrane review evaluating oral vitamin B12 supplementation during pregnancy—including five randomized controlled trials and nearly 1000 participants—did not demonstrate consistent evidence of long-term neurocognitive benefit [12]. Although one trial reported improved language expression at 30 months among offspring of supplemented mothers, overall findings remain inconclusive, likely due to small sample sizes, heterogeneous supplementation protocols, and variable neurodevelopmental assessment tools [165,166].

Folate (vitamin B9) is essential for DNA synthesis and neural tube closure. Experimental studies in animal models have shown that maternal folic acid supplementation enhances neurogenesis and synaptogenesis [167]. Its role in preventing neural tube defects is well established; however, evidence linking folate supplementation to broader neurodevelopmental outcomes remains less consistent [168].

Vitamin D also contributes to neurodevelopment by modulating neurogenesis, myelination, and inflammatory pathways via its receptor-mediated actions [169]. Although observational studies have associated low maternal vitamin D levels with adverse neurodevelopmental outcomes, current evidence remains heterogeneous and insufficient to establish causality [170,171].

Iodine is indispensable for thyroid hormone synthesis and fetal brain development. Maternal iodine deficiency is associated with severe neurodevelopmental impairment and reduced intelligence quotient (IQ) in offspring [172].

Other trace elements are also involved in neurodevelopmental processes. Selenium participates in selenoprotein synthesis, thyroid hormone metabolism, and myelination [173]. A Danish cohort study reported an association between low maternal selenium levels and increased risk of attention-deficit/hyperactivity disorder (ADHD) in children; however, evidence remains limited [174]. Zinc plays a critical role in neuronal migration, differentiation, and myelination [175]. Higher maternal zinc intake—particularly during the third trimester—has been associated with improved white matter maturation and better cognitive performance in early childhood [176].

Iron is essential for oxygen transport and numerous enzymatic reactions involved in brain development. Although maternal iron deficiency has been linked to adverse neurodevelopmental and behavioral outcomes, findings are inconsistent. A systematic review reported only a weak association between third-trimester iron deficiency and later neurocognitive impairment [177,178].

Omega-3 fatty acids, particularly DHA and EPA, are integral components of neuronal membranes and support neurogenesis, synaptic plasticity, and myelination. Omega-3 deficiency during pregnancy has been associated with altered hippocampal development and reduced neuronal size in experimental models [163]. A systematic review of randomized trials found that omega-3 supplementation during pregnancy was associated with improved cognitive outcomes in offspring in five out of eight studies analyzed [179]. Beyond neurodevelopment, omega-3 fatty acids also contribute to long-term cardiovascular health [180].

### 7.2. Influence of Maternal Nutrition on Obesity and Metabolic Syndrome Risk

The period from conception through the first two years of life—the so-called “first 1000 days”—is critical for metabolic programming. Maternal diet and nutritional status during this window can substantially influence the offspring’s risk of obesity, metabolic syndrome, and other NCDs.

Maternal obesity during pregnancy has been associated not only with adverse metabolic outcomes but also with increased risk of neurodevelopmental disorders, including attention-deficit disorders and autism spectrum conditions [181]. Obesity is a multifactorial disease, and increasing evidence supports the contribution of intrauterine exposures to its pathogenesis [182].

Excess fetal exposure to nutrients and lipids may induce long-lasting epigenetic modifications, including altered DNA methylation patterns, leading to metabolic programming that predisposes offspring to insulin resistance, adiposity, and obesity later in life [183]. Moreover, some epigenetic changes may persist across generations. Maternal obesity is also characterized by chronic low-grade inflammation, which may impair placental function and alter nutrient transport, further influencing fetal metabolic development [184].

Conversely, maternal undernutrition can also increase the risk of metabolic disease in offspring. The “thrifty phenotype” hypothesis proposed by Hales and Barker suggests that fetal adaptations to nutrient scarcity promote energy conservation. While advantageous in nutrient-poor environments, such adaptations may predispose individuals to visceral obesity, insulin resistance, and metabolic syndrome when exposed to nutrient-rich postnatal environments [3].

Maternal diet may also influence offspring lipid metabolism and future cardiovascular risk, including the development of hypercholesterolemia [185]. These findings underscore the importance of balanced maternal nutrition as a strategy for primordial prevention of cardiometabolic disease beginning in utero.

### 7.3. Effects of Maternal Nutrition on the Microbiota

Growing evidence highlights the fundamental role of the gut microbiome in metabolic, immune, and neurological health. Alterations in early-life microbial colonization may have lasting consequences, increasing susceptibility to metabolic and immune-mediated diseases [186].

Although neonatal microbiota composition is influenced by multiple factors—including mode of delivery, breastfeeding, complementary feeding practices, and antibiotic exposure—maternal diet during pregnancy also plays a significant role. Diets rich in dietary fiber have been associated with greater microbial diversity and increased abundance of beneficial bacterial species, potentially promoting favorable metabolic and immune programming in the offspring [187,188].

The main long-term effects of maternal nutrition during pregnancy on offspring risk of non-communicable diseases are summarized in Table 3.

## 8. Limitations and Strengths of the Study

Narrative reviews are perfect for giving a wide background, pointing out research gaps, and examining emergent themes since they offer a flexible, thorough, and in-depth exploration of a subject. Their main strength is their ability to synthesize a wide range of literature, including theoretical and, occasionally, conceptual work, to provide fresh viewpoints and ideas. Narrative reviews, however, are constrained by their high subjectivity, potential for selection bias, and lack of reproducibility, which frequently results in conclusions that are not representative. They are less appropriate for evidence-based decisions or policy because, in contrast to systematic reviews, they lack rigorous, transparent processes for searching and evaluating literature. Subjectivity and bias, lack of structure and reproducibility, non-exhaustive search, and qualitative focus are some of the main drawbacks.

We believe that the major limitation of current evidence is the lack of data on nutrition of child-bearing-age women. Pregnancy offers a special window of opportunity to encourage healthier and more sustainable eating habits since women are more likely to be strongly motivated to improve their health and to see medical specialists more frequently during this time. Healthcare professionals must have culturally relevant, easily accessible, and evidence-based tools to facilitate perinatal discussions about nutrition, supplements, exercise, and body weight if they are to properly take advantage of this opportunity. In order to reduce health disparities, enhance long-term wellbeing, and have a beneficial impact on the health of future generations, it is crucial to provide appropriate care during the periconceptional and early pregnancy periods.

The main strength of our narrative review is that it focuses on the dyad mother–fetus and then mother–newborn and child as a unique entity in terms of health outcomes related to nutritional issues. Our perspective is that of using nutrition as a health voucher for both mother and fetus or mother and child.

## 9. Conclusions

The Barker hypothesis and the broader DOHaD framework underscore the critical role of early-life nutrition—including the intrauterine period—in shaping long-term health trajectories. Suboptimal maternal nutrition during pregnancy may increase the offspring’s risk of developing metabolic syndrome, encompassing obesity, type 2 diabetes, insulin resistance, hypertension, and dyslipidemia, as well as related complications such as coronary heart disease and stroke. Moreover, exposure to energy-dense Western dietary patterns, particularly when followed by rapid postnatal growth associated with high caloric intake, may further amplify the cardio-metabolic risk.

Collectively, current evidence highlights that both quantity and quality of maternal nutrition during pregnancy are fundamental determinants of fetal growth, organ development, and long-term disease susceptibility. Ensuring adequate and balanced nutritional intake during this critical window represents a key strategy for optimizing neonatal outcomes and promoting lifelong health, beginning from the earliest stages of development. Future studies should focus on women’s nutrition as a continuum starting from childhood up to pregnancy and lactation, so as to build the adequate macronutrients and micronutrients stores and to help root healthy nutritional patterns. In this context, nutrition can act as a bridge form the concept of “to cure” to that of “to care”.

## Figures and Tables

**Table 1:** Main dietary recommendations during pregnancy, adjusted from [25,26,27,28,29,30].

**Category**	**Recommendation**	**Notes/Rationale**
**General Principles**	Adequate energy intake with modest progressive increase. Nutrient-rich diet based on minimally processed, varied foods and selected micronutrient supplementation. Regular physical activity, avoid smoking and alcohol.	Individualized counseling based on pre-pregnancy BMI, physical activity, dietary habits, and socio-cultural context. Monitor gestational weight gain (GWG).
**Energy Requirements**	Additional energy requirement in 1st trimester (T): ≈70 kcal/day. Additional energy requirement in 2nd T: ≈260 kcal/day. Additional energy requirement in 3rd T and first 6 months of breastfeeding: ≈500 kcal/day.	Adjust according to BMI and GWG targets.
***Macronutrients***		
**Carbohydrates**	45–65% of total energy; ≥175 g/day. ≥50% from whole grains; <10–15% from simple sugars, and correlated to physical activity level	Ensures adequate maternal/fetal brain glucose needs.
	Fibre-rich foods: 5 portions/day of legumes, fruit, vegetables, nuts.	Supports glycemic control.
**Protein**	Intake should be kept within 25% of total energy. 1.1 g/kg/day after 1st trimester. ≈54 g/die +1 g (1st T); +8 g (2nd T); +26 g (3rd T).	Plant + animal source OR combined plant proteins to enhance overall protein quality.
		Balanced intake reduces the risk of stillbirth, LBW, and SGA.
**Fats**	20–35% of total energy. Prioritize unsaturated fats; limit saturated and trans fats. DHA requirement increases by 100–200 mg/day. Choose low-mercury fish (e.g., salmon, cod).	Fundamental structural constituents of cellular membranes and play critical roles in tissue development.
		n-3 PUFAs supplementation reduces preterm birth and LBW (evidence evolving).
***Micronutrients***		
**Folic Acid**	Routine supplementation: 400 µg/day starting ≥30 days before conception → 600 µg/day during at least 1st trimester. 4–5 mg/day in high-risk women (history of neural tube defects, malabsorption, etc.)	Essential for DNA and RNA synthesis, methylation of homocysteine to methionine, and amino acid metabolism. Prevention of neural tube defects, anemia, LBW; additional congenital heart protection suggested.
**Iron**	Routine supplementation: 30–60 mg/day (WHO/FIGO). No routine supplementation (NICE/SINU). Hb < 11 g/dL in 1st T, <10.5 g/dL after 28 weeks should be assessed and managed appropriately. Iron-deficiency anemia treatment: 60–120 mg/day oral iron.	Enhance absorption of plant-derived iron with vitamin C-rich foods. Avoid concurrent intake of tea/coffee/calcium-rich foods.
		Essential component of hemoglobin, myoglobin; needed for several oxidoreductive enzymes. Deficiency is linked to impaired fetal growth, LBW, and preterm birth. Possible cardiometabolic consequences for the offspring.
**Calcium**	Reference intake: 1.1 g/day (LARN V). Supplementation with 1.5–2.0 g/day from the 20th week until the end of pregnancy if low dietary intake or low serum levels	Essential for skeletal fetal development. Selected supplementation to prevent the risk of hypertension, pre-eclampsia, and preterm birth.
**Vitamin D**	Supplementation: 400 IU/day to all women (FIGO/NICE); 200 IU/day if deficiency suspected (WHO); selective supplementation only in women at high risk of hypovitaminosis due to personal/environmental factors or dietary deficiencies (SIGO/AOGOI/GUI)	Monitor levels during pregnancy. Deficiency has been associated with increased risk of preterm birth, SGA or LBW infants, recurrent miscarriage, and gestational diabetes mellitus.
**Vitamin A**	Not routinely recommended. Supplementation only in areas where deficiency is common or in HIV + women	Reduces maternal night blindness and maternal anemia
**Vitamin B12**	Not routinely recommended. Supplementation is advised if deficient or high risk (e.g., vegetarian/vegan dietary patterns)	Important for one-carbon metabolism, DNA synthesis, erythropoiesis, and neurodevelopment. Deficiency has been associated with LBW, neural tube defects, and impaired neurodevelopment.
**Iodine**	Adequate intake: 200 µg/day (EFSA/LARN); ≈250 µg/day (WHO). Supplementation with 150–250 µg of iodine is recommended in women living in known mild-moderate iodine-deficient areas. Supplementation with 50 µg in countries with successful salt iodization programs.	Supplementation should start preconception if possible. Special care in case of intestinal malabsorption, vegan diets, and low-carbohydrate diets.
		Important for thyroid-hormone production. Deficiency is linked to miscarriages, perinatal mortality, congenital defects, and neurodevelopment.
**Zinc & Others**	Supplementation of zinc and other minerals (Mg, Na, K, Se, Cu) and vitamins (B6, C, E) is not routinely recommended if the diet is balanced.	
***Others***		
**Alcohol**	Refrain from alcohol consumption during the peri-conception period and 1st trimester, and subsequently refrain or, in any case, limit intake to no more than 2 glasses (one glass = 125 mL) of red wine per week.	Associated with risk of FASD (congenital defects, growth restriction, distinctive facial features, neurodevelopmental problems), miscarriage, preterm birth, SGA
**Caffeine**	It is recommended to limit caffeine consumption to no more than 200 mg per day (2 small cups).	Safety limits are not yet clearly defined.
		High intake linked to miscarriage/LBW.

**Table 2:** Possible benefits and adverse effects related to vegan diet in pregnancy (adapted from [104,105,106,107,108,109]).

Pros	Cons	Important Nutrients to Track
Decreased Complication Risk: There is a decreased chance of gestational diabetes. Healthy Weight Gain: Less chance of gaining too much weight when pregnant. Reduced Blood Pressure: Pregnancy-related hypertension may be less likely. Better Digestion: Consuming more fiber can help with common problems like constipation. High consumption of vitamins, fiber, and antioxidants from fruits, vegetables, and legumes is known as nutrient density.	Vitamin B12, iron, iodine, calcium, vitamin D, and omega-3 fatty acids may be deficient, requiring monitoring and supplementation. Risk of Low Birth Weight: Strict vegan diets have been linked in several studies to a higher risk of low birth weight or small-for-gestational-age babies. Careful Planning Is Required: requires strict monitoring of consumption of micronutrients and protein to guarantee fetal growth. Possible Vitamin B12 Deficiency: If not supplemented, this could result in serious developmental problems	Supplementing with vitamin B12 is necessary for the growth of nerves. Iron is essential for preventing anemia and can be found in spinach, beans, and lentils. Flax seeds and walnuts contain omega-3 (DHA/EPA), which is essential for brain development. Protein: Found in quinoa, almonds, pulsed and tofu.

**Table 3:** Main effects of maternal nutrition during pregnancy on non-communicable diseases in the offspring. (Abbreviations used: DOHaD Developmental Origin of Health and Disease, SGA Small for Gestational Age, LBW Low Birth Weight).

Maternal Nutritional Exposure	Main Offspring Outcome	Implications for NCD Risk	Study Design	Reference *
Low birth weight	Increased adult cardiovascular and metabolic disease	Foundation of DOHaD; fetal programming of cardiometabolic risk	Observational epidemiological studies	Barker et al., 1986–1993 [3,4,5,6,7]
Altered fetal nutrition	Increased adult cardiometabolic risk	Support for fetal origins of chronic disease	Narrative review	Godfrey & Barker, 2000 [3]
Maternal undernutrition (“thrifty phenotype”)	Insulin resistance, visceral obesity	Mechanistic link to metabolic syndrome	Theoretical + epidemiological evidence	Godfrey& Barker, 2000 [3]
Balanced energy–protein supplementation	Reduced SGA, LBW, stillbirth	Potential reduction in later cardiometabolic disease risk	Systematic review	Keats et al., 2021 [8]
Healthy vs. Western dietary patterns	Healthy diet: ↓ SGA & preterm; Western: ↑ SGA & complications	Western pattern linked to increased cardiometabolic risk	Systematic review and meta-analysis	Abdollahi et al., 2021 [17]
Maternal iron status	Altered birth weight; possible vascular effects	Potential long-term cardiometabolic implications	Prospective cohort	Alwan et al., 2015 [11]
Vitamin B12 supplementation	Inconclusive neurocognitive benefit	Insufficient evidence for long-term neuroprotection	Cochrane systematic review	Finkelstein et al., 2024 [12]
Omega-3 deficiency	Altered hippocampal development	Potential increased neurodevelopmental risk	Experimental study	Cortés-Albornoz et al., 2021 [163]
High ultra-processed food intake	Increased neonatal body fat	Programming toward childhood obesity	Observational study	Rohatgi et al., 2017 [130]
Overall maternal diet quality	Reduced infant atopic dermatitis	Protective effect against immune-mediated diseases	Cohort study	Li et al., 2023 [137]
Early-life nutritional exposures	Epigenetic modifications	Transgenerational transmission of NCD risk	Mechanistic review	Gluckman et al., 2008 [22]

* The articles listed in the table that fall outside the search range specified in the Methods section were included because they are considered historical studies.

## Data Availability

Not applicable.

## Abbreviations

The following abbreviations are used in this manuscript:

AA	Arachidonic Acid
ADHD	Attention Deficit Hyperactivity Disorder
AI	Adequate Intake
BMI	Body Mass Index
DHA	DocosaHexaenoic Acid
DOHaD	Developmental Origins of Health and Disease
EFSA	European Food Safety Authority
EPA	Eicosapentaenoic Acid
FASD	foetal alcohol spectrum disorders
FDA	Food and Drugs Administration
FIGO	Federazione Italiana Ginecologi e Ostetrici
GDM	Gestational Diabetes Mellitus
GWG	Gestational Weight Gain
KD	Ketogenic Diet
IGF-1R	Insulin-like Growth Factor-1R
IQ	Intelligence Quotient
LARN	Livelli di Assunzione di Riferimento di Nutrienti ed energia per la popolazione italiana
LGA	Large for Gestational Age
MedDiet	Mediterranean Diet
NICE	National Institute for Health and Care Excellence
PBDs	Plant Based Diets
PCOs	PolyCistic Ovary syndrome
PDCAAS	Protein Digestibility-Corrected Amino Acid Score
PUFAs	polyunsaturated fatty Acids
SGA	Small for Gestational Age
SINU	Società Italiana di Nutrizione Umana
TC	Total Cholesterol
UPFs	Ultra-Processed Foods
WHO	World Health Organization
